# Extending the Traceability of Dynamic Calibration to the High-Pressure Regime Using a Shock Tube

**DOI:** 10.3390/s25082453

**Published:** 2025-04-13

**Authors:** Eynas Amer, Gustav Jönsson, Olle Penttinen, Fredrik Arrhén

**Affiliations:** Measurement Science and Technology, RISE Research Institutes of Sweden, 504 62 Borås, Sweden; gustav.jonsson@ri.se (G.J.); olle.penttinen@ri.se (O.P.); fredrik.arrhen@ri.se (F.A.)

**Keywords:** dynamic pressure, dynamic calibration, shock tube, dynamic sensors, measurement uncertainty

## Abstract

In this paper, a development of the shock tube at RISE, the National Metrology Institute of Sweden, to extend its capability to the high-pressure regime is presented. The shock tube was developed to be operated in three different configurations: conventional, with an amplification system and with a converging cone. In the conventional and with the amplification system, the well-established shock tube analytical solution was used to calculate the reference pressure, while in the converging cone, a numerical simulation was applied. To demonstrate the capabilities and limitations of each configuration, a device under test (DUT) was characterized. The results show a good agreement in the DUT dynamic response calculated using the three configurations in the overlap regions between them. The uncertainty in measurements was estimated for each configuration. The three configurations complement each other to reach a pressure range from 0.1 MPa to 25 MPa and a frequency range from 0.5 kHz to 500 kHz.

## 1. Introduction

In correspondence with increasing focus on sustainability, dynamic pressure measurements play a vital role in several demanding fields such as automotive, maritime, aerospace, hydraulics, materials testing, combustion and explosion safety [1,2,3,4]. The accuracy and reliability of the measurements are essential to achieve process control and system optimization. This emphasizes the needs for the traceability to the International System of Units (SI) [5] for dynamic pressure measurements which in turn requires well-established standards and methods [6,7,8]. Substantial development of dynamic pressure primary and secondary standards is already in progress, with drop-weight devices [6,9] and shock tubes [10,11,12,13] as major candidates.

Drop-weight devices utilize the impact of a dropping weight of known mass on a liquid-filled chamber and can generate half-sine pressure pulses in the range from 5 MPa to 500 MPa with an estimated uncertainty of around 2–3%. While it is suitable for generating high pressures, it is limited in the lower ranges. Furthermore, it has a limited bandwidth, typically less than 1 kHz. Shock tubes are devices capable of generating a rapid step-like pressure with a risetime well below 1 μs, thus exciting frequencies in the megahertz range. A conventional shock tube can generate a moving shock wave of desired strength, forming a reference pressure step upon reflection from its end wall. The amplitude of the pressure step can be calculated using the well-established shock tube analytical solution. The reference amplitude can be calculated with a relatively low uncertainty of 1–2%. However, the upper limit of the realized reference pressure is restricted by the material strength used in the construction of the shock tube and the opening mechanism. The shock tube at RISE can withstand static pressures up to 2 MPa, significantly limiting its capability.

To extend the capability of the shock tube to a high-pressure regime, two novel technologies of amplifying the generated reference pressure have been implemented. The first one is based on using a smooth area reduction technique, converging cone, that was developed by a research group at the KTH Royal Institute of Technology, Sweden [14]. The converging cone transforms the incident plane shock to a spherical shock wave which, upon convergence, accelerates the shock wave, thereby increasing its energy density. Using a device based on the same principle of the RISE shock tube, the realized reference pressure was increased almost 15 times compared to using the conventional shock tube under similar initial conditions. However, the realized pressure no longer had a step-like profile but a blast profile. The amplification was not sustained at the desired level, instead it dropped rather immediately due to the expansion of the reflected wave. As a result, the analytical solution was not applicable, and mathematical modelling and numerical simulations were applied to calculate the reference pressure. The dependence on simulations to predict the generated reference pressure limits the capability of the shock tube with a converging cone as a primary standard in this pressure range, and the validation of the method is mandatory.

The second method used to amplify the reference pressure is based on the development of the converging technology to generate a step-like profile while preserving the amplification. To retrieve the step-like profile, a new amplification system which comprised an extension tube attached to a converging cone through a smooth transition was developed. The new system can generate an amplified step-like reference pressure (up to 7 MPa) that can be maintained up to 0.2–0.3 ms. Since the step-like profile was retrieved, the shock tube analytical solution was used to calculate the generated reference pressure.

The purpose of this work is to present the development of effective methods of pressure amplification using new technologies. The shock tube was operated in three configurations, a conventional shock tube, shock tube with a converging cone and shock tube with an amplification system consisting of an extension tube smoothly attached to a converging cone. For the conventional shock tube and for the amplification system configurations, the well-established shock tube analytical solution was used to calculate the reference pressure, while for the converging cone configuration, the reference pressure was estimated using a numerical simulation as mentioned above. To demonstrate the capabilities and the limitations of each configuration, a device under test (DUT) was characterized using the three configurations. The results from the three configurations in terms of the sensitivity of the DUT in the frequency domain were calculated and compared. Furthermore, the uncertainty budget was assessed for each configuration.

## 2. Shock Tube Theory

**Figure 1:**
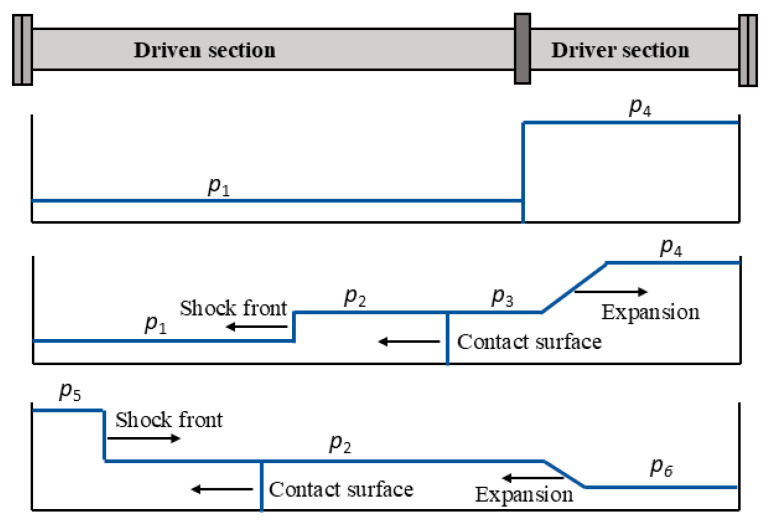
An illustration of the shock wave propagation, *p*_1_: the initial driven gas pressure, *p*_4_: the initial driver gas pressure, *p*_2_: the pressure behind the incident shock front, *p*_3_: the pressure behind the expansion wave*, p*_5_: the pressure behind the reflected shock front and *p*_6_: the pressure behind the reflected expansion wave.

Shock tubes are gas dynamic instruments used for studies of high-temperature gases in physics and chemistry [15,16]. The shock tube consists of two parts: a driven section, filled with a driven gas of low pressure, *p*_1_, and a driver section that contains a driver gas of high pressure, *p*_4_, which are separated by an opening mechanism (diaphragm or fast-opening valve). When the separation between the two parts is opened, an incident shock wave propagates in the driven section toward its end-wall plate, whereas an expansion wave propagates into the driver section. As the incident shock wave propagates, it increases the pressure of the gas behind it (region 2) and induces mass motion with velocity *U*_2_. The interface between the driven and the driver gases is called the contact surface, and across it, the pressure and velocity are preserved, whereby *p*_2_ = *p*_3_ and *U*_2_ = *U*_3_. When the incident shock wave impinges on the end-wall plate, it reflects with pressure *p*_5_ behind it. The expansion wave propagates into the driver section, smoothly decreasing the pressure in region 4 to a lower value of *p*_3_ behind it. The expansion wave reflects upon the end-wall plate of the driver section with pressure *p*_6_ behind it. The reflected expansion wave propagates toward the driven section and interacts with the reflected shock wave. Figure 1 illustrates the propagation of the shock wave at different stages. The constant pressure time before the interaction between the expansion and the reflected shock wave is of importance, and several research attempts have been performed to extend this time [17,18]. The pressure, temperature and mass velocity of the shock wave propagating in a calorically perfect gas (having constant specific heats with respect to temperature) can be calculated from the measured shock propagation speed and the static initial conditions using a 1-D transport model [19,20].

The Mach number *M* of the incident shock front is the ratio of its propagation speed in the laboratory frame *v* and the speed of sound in the undisturbed driven gas $a_{1}=\sqrt{\gamma_{1}R_{1}T_{1}}$, where *γ*_1_ is the specific heat ratio, *R*_1_ is the specific gas constant and *T*_1_ is the measured initial absolute temperature of the driven gas. From the calculated Mach number and the initial parameters of the driven gas, its pressure (*p*_1_) and its temperature (*T*_1_), the gas parameters behind the incident shock wave (region 2) can be calculated. The gas pressure (*p*_2_), the mass velocity (*U*_2_) and the temperature (*T*_2_) behind the incident shock wave can be calculated according to the following equations:

(1) $$p_{2}=p_{1}\left(1+\left(\frac{2\cdot \gamma_{1}}{\gamma_{1}+1}\right)\left(M^{2}-1\right)\right)$$

(2) $$U_{2}=\frac{a_{1}}{\gamma_{1}}\left(\frac{p_{2}}{p_{1}}-1\right){\left(\frac{\frac{2\gamma_{1}}{\gamma_{1}+1}}{\frac{p_{2}}{p_{1}}+\frac{\gamma_{1}-1}{\gamma_{1}+1}}\right)}^{1/2}$$

(3) $$T_{2}=T_{1}\left(\frac{p_{2}}{p_{1}}\right)\left(\frac{\frac{\gamma_{1}+1}{\gamma_{1}-1}+\frac{p_{2}}{p_{1}}}{1+\frac{\gamma_{1}+1}{\gamma_{1}-1}\left(\frac{p_{2}}{p_{1}}\right)}\right)$$

Furthermore, the pressure behind the reflected shock front at the end-wall plate (*p*_5_) can be calculated as

(4) $$p_{5}=p_{2}\left(\frac{\left(\frac{\gamma_{1}+1}{\gamma_{1}-1}+2\right)\left(\frac{p_{2}}{p_{1}}-1\right)}{\frac{\gamma_{1}+1}{\gamma_{1}-1}+\frac{p_{2}}{p_{1}}}\right)$$

Finally, the amplitude of the reference step pressure Δ*p* is calculated as

(5) $$\Delta p=p_{5}-p_{1}$$

## 3. Experimental Setup and Procedures

The shock tube at RISE was developed to be operated in three different configurations: conventional, with a converging cone and with an amplification system comprising an extension tube attached to a converging cone through a smooth transition. In the following section, the three operational configurations and the procedures to calculate the corresponding reference pressure will be presented. Moreover, the method to calculate the dynamic response of a device under test in terms of its sensitivity in the frequency domain will be described.

In all experiments, Ar (99.999%) was used as the driven gas, while the driver gases were Ar (99.999%) and He (99.999%), respectively. The DUT used in this study was a PCB 113B22 piezoelectric sensor with a PCB 483C05 signal conditioner. The sensor has a measurement range of up to 34.47 MPa (gauge) and a natural frequency higher than 500 kHz. In all experiments, the DUT was flush mounted at the centre of the end-wall plate of the respective configurations. The output signal from the DUT and the side-wall sensors were acquired using an 8 channel 12 bit 60 MS/s channel oscilloscope (PXI-5105, NI, Austin, TX, USA) with a maximum sampling size of 10^6^ samples. The LabVIEW 2019 software was used for data acquisition. In this work the sampling rate and size were 3 MS/s and 10^5^, respectively.

### 3.1. The Conventional Shock Tube

**Figure 2:**
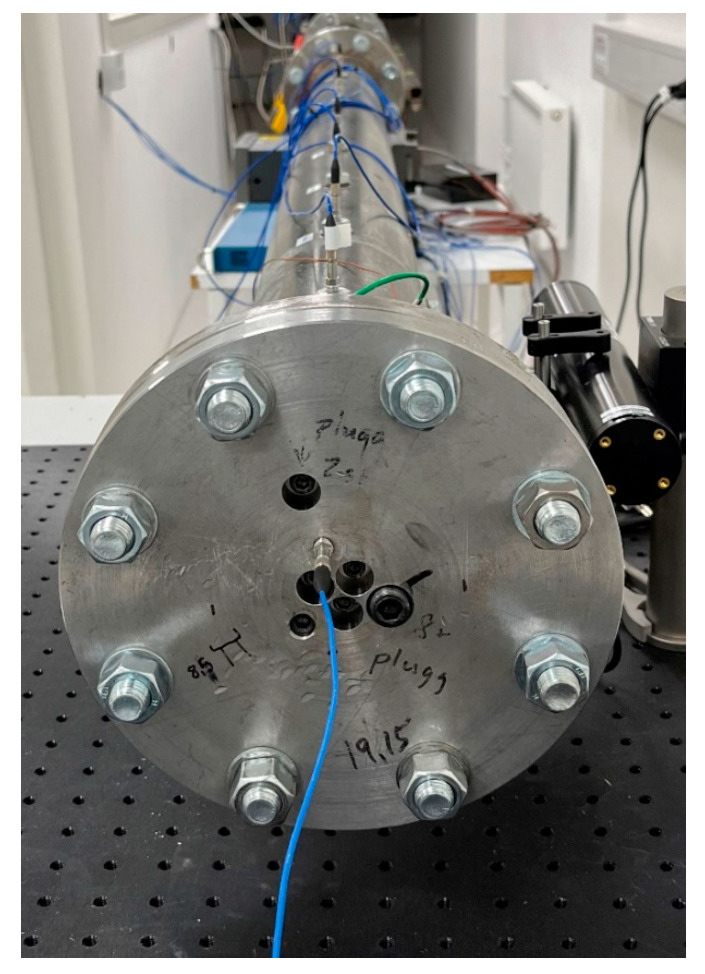
A photo of the conventional shock tube.

Figure 2 shows a photo of the conventional shock tube. A detailed description of the shock tube can be found in our previous publication [10]. The amplitude of the step pressure was calculated using Equations (1), (4) and (5) in Section 2. We used the same method as in our previous publication [10]. To keep this publication original and avoid repetition, we do not represent it here.

### 3.2. The Shock Tube with a Converging Cone

In the following section, the design criteria of the converging cone and the numerical simulation for the reference pressure calculation will be described.

#### 3.2.1. The Configuration of the Converging Cone

**Figure 3:**
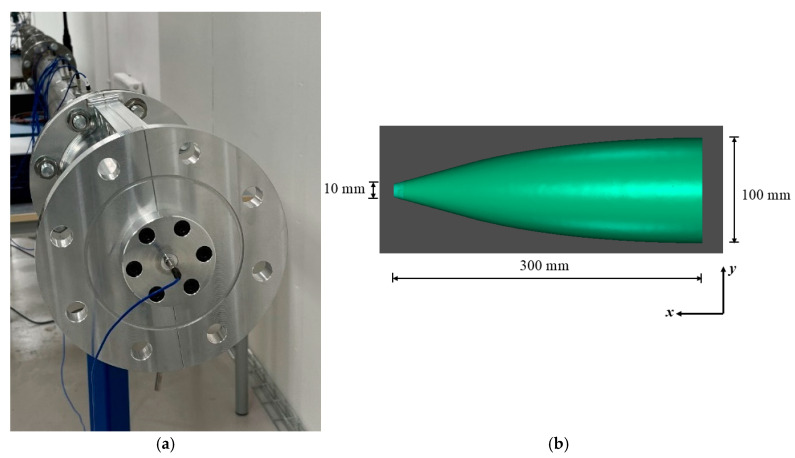
(**a**) A photo of the shock tube with the converging cone. (**b**) The internal geometry of the converging cone.

A converging section of a cone-like shape was directly connected to the straight driven section, as shown in Figure 3a. The converging cone amplifies the generated reference pressure using the smooth area reduction technique. The cone is a 300 mm long section with a diameter that decreases smoothly from 100 mm at the inlet to 10 mm at the outlet, as shown in Figure 3b. The converging cone transforms the incident plane shock wave to a spherical shock wave and accelerates it along its length, thereby increasing its energy density. In order to fulfil the requirement that the shock foot remains normal to the wall without the creation of the Mach stem during its convergence, the design of the cone was based on the criteria developed by Kjellander et al. [21,22].

(6) $$\left\{\begin{array}{c} x=Asin\;\theta ,\\ y=B-R\left(1-cos\;\theta \right) \end{array}\right.$$

where 0 ≤ *θ* ≤ 0.27π, A = 300.7 mm, B = 50 mm and R = 71.625 mm. For *θ* > 0.27π, a tangent using the three previous points was calculated.

#### 3.2.2. Reference Pressure Calculation for the Converging Cone Configuration

Since the realized reference pressure generated by using the converging cone no longer had a step-like profile but a blast profile, the analytical solution for the reference pressure calculation is not applicable. Instead, the reflected pressure at the tip of the converging cone was determined in the temporal domain via numerical simulations. The input parameters to the numerical simulations comprise the measured geometry of the cone and the gas parameters calculated from Equations (1)–(3). For numerical simulations, the OpenFOAM-7 2019 software with the rhoCentralFOAM solver was used. As described in the OpenFOAM user guide, the rhoCentralFOAM solver is a transient solver capable of capturing compressibility, heat transfer and shocks [23]. The solver equations are based on the central-upwind schemes of Kurganov and Tadmor [24]. A laminar simulation type was applied to avoid dealing with setting turbulence properties.

**Figure 4:**
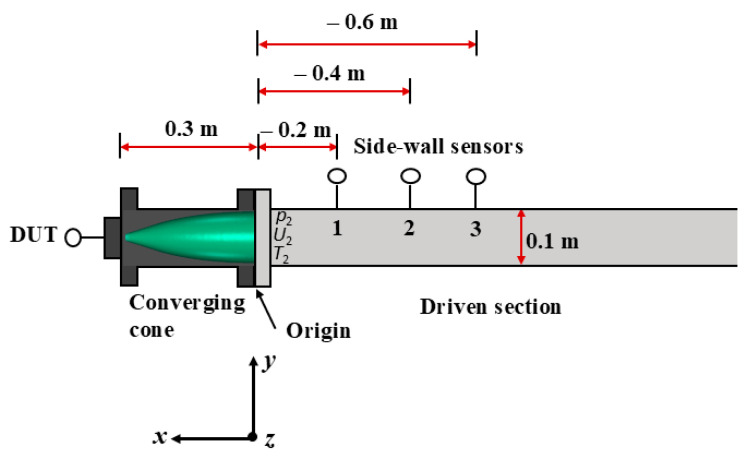
A schematic illustration of the dimensions used in the numerical simulation.

**Figure 5:**
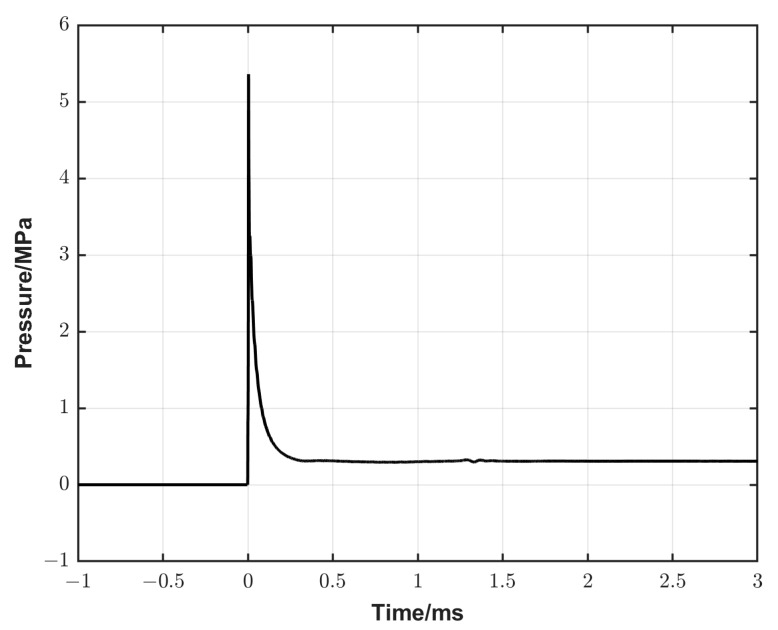
A typical reference pressure generated by the converging cone configuration calculated using numerical simulation at a nominal pressure of 5.3 MPa.

The dimensions of the simulation domain were as follows: in x-direction, from −0.5 m to 0.3 m; in y-direction, from 0 m to 0.05 m; and in z-direction, from −0.0022 m to 0.0022 m. An illustration of the dimensions used in the simulation is presented in Figure 4. The domain was assumed to be axisymmetric, hence only a “2D” slice with a maximum thickness of 4.4 mm in the wall of the adjacent cells of the straight pipe wall was simulated. The average computational cell size was 23 mm^3^, ranging from a maximum of 103 mm³ at the wall of the straight section to a minimum of 0.015 mm^3^ at the centre of the cone where the reference pressure profile was sampled. We aimed to establish a smooth transition from a structured mesh in the straight section to an unstructured tetrahedral mesh in the converging section. Constraints put on the unstructured mesh resulted in a continuous decrease in cell sizes towards the DUT position. The cells closest to the DUT had a length of 1 mm in the x direction and 2 mm in the y direction. Mesh studies showed that a further decrease in cell size had negligible effects on simulation results. The initial conditions (ICs) and the boundary conditions (BCs) of different parameters used in the simulation are listed in Table 1. For efficient post-processing, virtual probes for *p*, *U* and *T* were inserted at the same locations as the physical probes (the side-wall sensors and the DUT). This enabled synchronization with the physical experiments as well as the comparison of shock amplitude versus shock front velocity. The simulation time is about 4 h at each pressure level. A typical reference pressure calculated using numerical simulation for the converging cone configuration at a nominal pressure of 5.3 MPa is shown in Figure 5.

### 3.3. The Shock Tube with an Amplification System

In this configuration, an amplification system consisting of an extension tube smoothly attached to a converging cone was connected to the driven section of the shock tube. The amplification system simultaneously amplifies the amplitude and preserves the step-like profile of the reference pressure. The step-like profile, in other words, means that there is a plateau of constant gas parameters behind the reflected shock wave. Since the shock relations are valid in non-accelerating flow, the reference pressure can be calculated analytically using the shock tube model.

#### 3.3.1. The Design of the Amplification System

The idea was to attach a uniform cross-section extension to the converging cone that simultaneously preserves the amplification and smoothly transitions the spherical shock wave into a non-accelerating plane shock wave. As the shock front travels at a constant velocity (after amplification), its speed can be measured at various positions using the time-of-arrival method.

**Figure 6:**
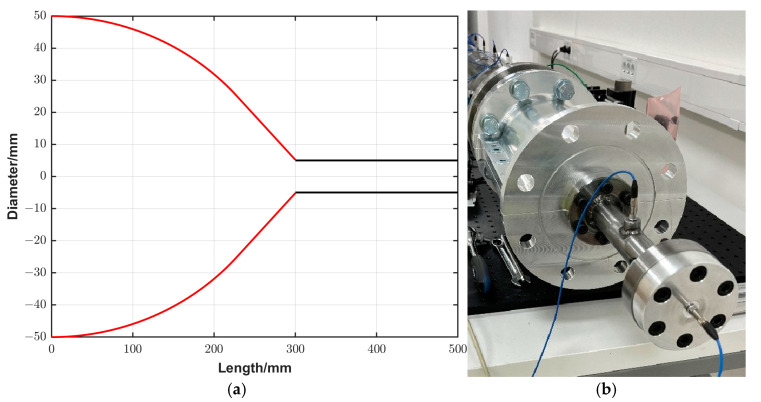
(**a**,**b**) An illustration and a photo, respectively, of the amplification system (first trial).

**Figure 7:**
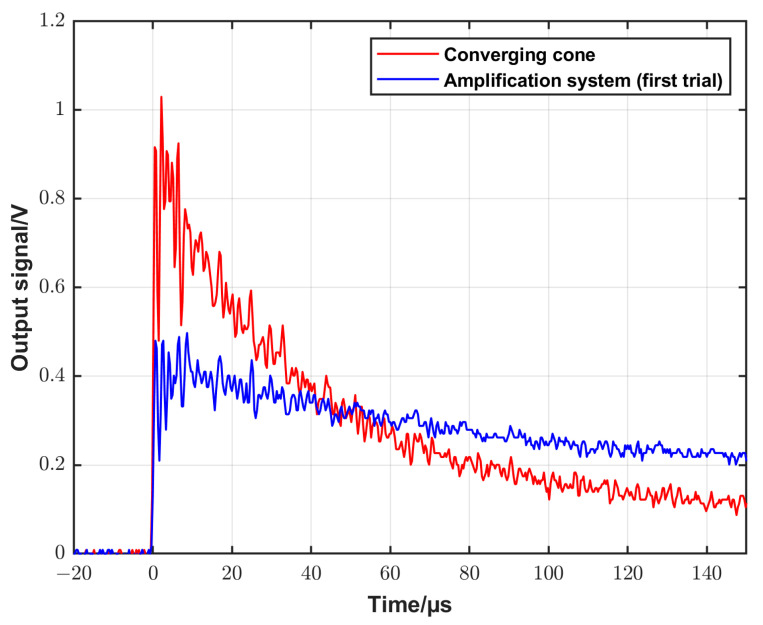
A comparison of the output signal of the DUT when using the converging cone and the amplification system (first trial) configurations, respectively, at the same initial conditions.

To achieve this purpose, an extension tube with a diameter of 10 mm (matching the outlet diameter of the cone) and a length of 200 mm was attached to the previously described converging cone (Section 3.2). The geometry of this configuration and a photo are shown in Figure 6a,b, respectively. This configuration will be called as an amplification system (first trial) in the following section. Measurements using this configuration and using only the converging cone at the same initial conditions were performed, and the recorded signals are presented in Figure 7.

**Figure 8:**
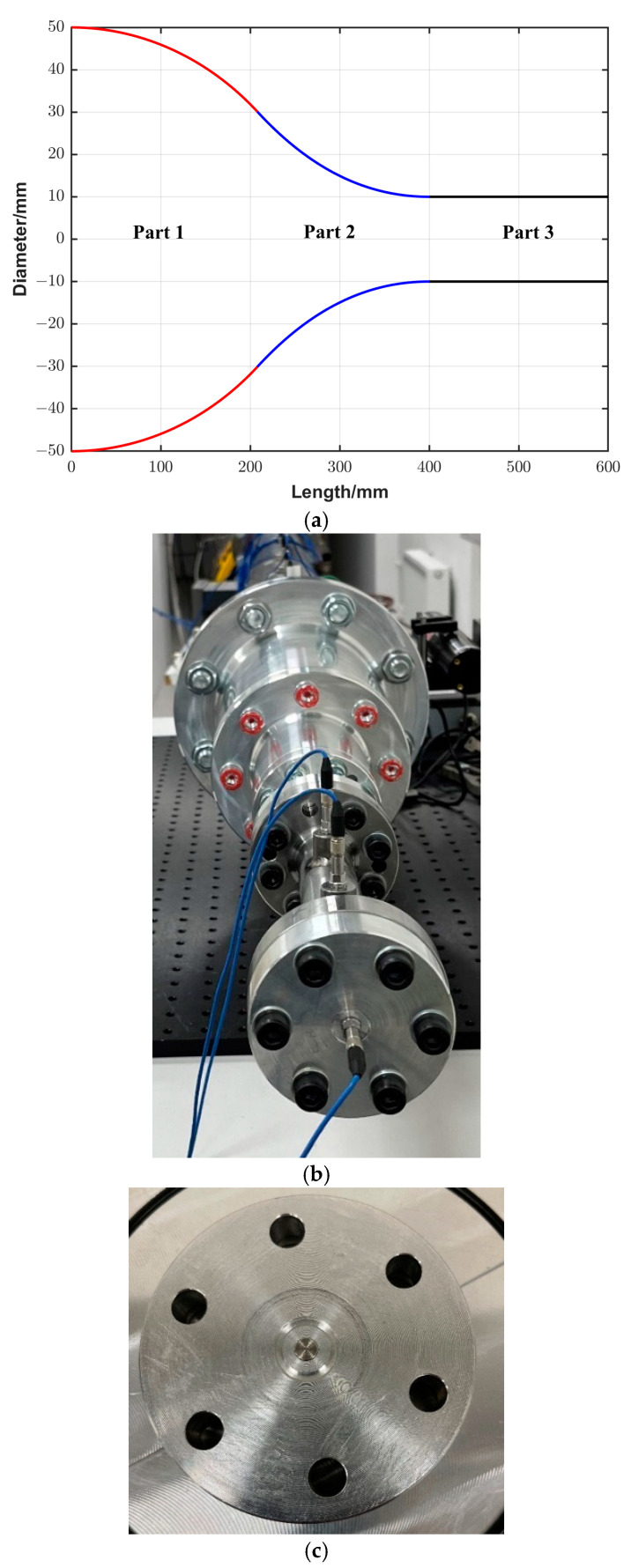
(**a**) The design of the amplification system comprising an extension tube smoothly attached to a converging cone. (**b**) A photo of the shock tube with the amplification system. (**c**) A photo of the backplate of the amplification system with the flush-mounted DUT.

**Figure 9:**
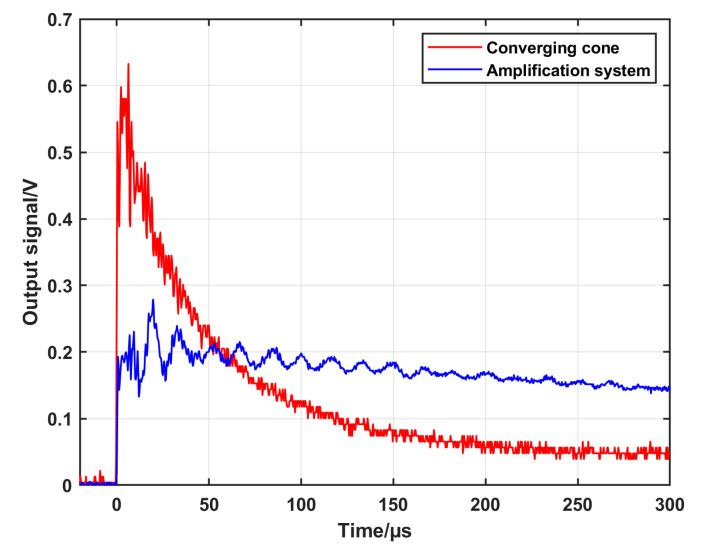
Typical output signals generated when using the converging cone and the amplification system configurations, respectively, at the same initial conditions.

The figure shows that the amplitude of the signal dropped to about half its value with the extension tube compared to when using only the converging cone, while the time of the constant signal increased from about 5 µs to about 20 µs, which in turn means that the purpose of using the extension tube was not reached. The amplitude decreased, and the constant pressure time was not long enough to retrieve the desired step-like shape. The reason for this is most likely due to the reflection at the walls due to the sharp transition between the cone outlet and the extension tube. To overcome this shortage, a new amplification system was developed. The new system consists of three parts. The first part is 208 mm long with a diameter that decreases smoothly from 100 mm at the inlet to 60.2 mm at the outlet according to Equation (6). The third part is an extension tube with a dimeter of 20 mm diameter and a length of 200 mm. An intermediate second part was designed by applying a smooth interpolation between the first and the third parts. Figure 8a,b show an illustration of the different parts of the system and a photo of the system attached to the driven section, respectively. Figure 8c shows the backplate of the amplification system with the flush-mounted DUT. To measure the speed of the shock wave in the extension tube, two side-wall piezoelectric sensors (PCB and 113B22) were mounted at well-defined positions of 60 mm and 140 mm, respectively, from the backplate. For comparison, the output signals were recorded when using the converging cone and the new amplification system configurations, respectively, at the same initial conditions. The recorded signals are displayed in Figure 9. It is seen that the constant signal time is about 200 µs, which is long enough to apply the analytical solution for reference pressure calculation. On the other hand, the amplitude of the generated signal is about one-third of the signal when using the converging cone. The reason for this decrease in amplitude is because the outlet diameter of the new system increased to 20 mm compared to 10 mm of the old cone. Also, the propagation distance along the extension tube causes losses in the energy of the generated signal.

#### 3.3.2. Reference Pressure Calculation

Since a plateau was detected when using the amplification system, the same analysis used in the conventional shock tube was applied. *v* was determined by fitting a quadratic polynomial to the time of arrival of the shock front at the positions of the two side-wall sensors and the end-wall sensor (see Figure 8b). All sensors are of the same model. The derivative of the position with respect to time at the end-wall plate position represents the shock propagation speed. The amplitude of the reference pressure was calculated using Equations (1), (4) and (5) in Section 2.

The characteristics of the four configurations of the shock tube tested in this study are summarized in Table 2. However, the amplification system (first trial) will be excluded from the Results Section, since it was an intermediate step towards the final design of the amplification system.

### 3.4. Dynamic Response Calculations

The dynamic response of the DUT in terms of its sensitivity in the frequency domain was calculaed using Fast Fourier Transform (FFT). The sensitivty is the ratio of the change in the DUT output to a change in the value of the measurand. (In our case, it is the reference pressure.) It is expressed in terms of voltage vs. pressure [8]. FFT was applied to both the recorded signal from the DUT and the corresponding calculated reference pressure using a hann window centred at the pressure discontinuity of the recorded signal. Sensitivity was calculated as the magnitude of the ratio between the FFT of the signal and the FFT of the coressponding reference. The sensitivity of the DUT at each pressure level was an average of three measurements. The full width at half the maximum (FWHM) of the Hann window was adjusted according to the constant pressure time of the corresponding reference (the conventional configuration and the amplification system). In the case of the conventional configuration, it was 1.7 ms and 1 ms when using Ar and He, respectively, as the driver gases. Since the plateau was relatively short in the case of using the amplification system, the FWHM was set to 0.6 ms. In the converging cone, an FWHM of 1 ms was applied.

## 4. Results

To demonstrate the capabilities and the limitations of the three configurations, a device under test (PCB 113B22 piezoelectric sensor with a PCB 483C05 signal conditioner) was characterized. The dynamic response of the DUT was calculated, and the uncertainty budget was assessed.

### 4.1. Characterization of the DUT

**Figure 10:**
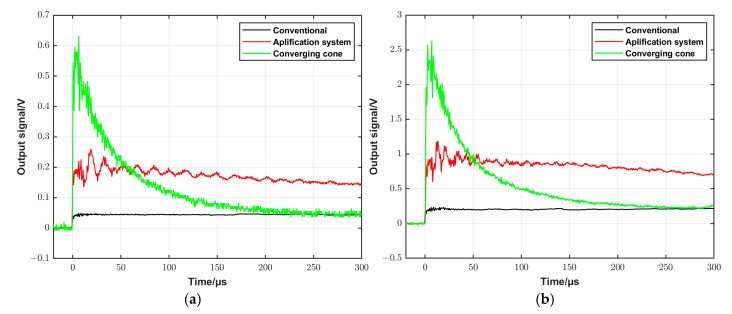
Typical output signals recorded by the DUT using the three configurations, namely the conventional, the amplification system, and the converging cone, respectively, at two different settings. (**a**) Ar was used as the driven and driver gas, with *p*_1_ = 100 kPa and *p*_4_ = 800 kPa. (**b**) Ar was used as the driven gas and He as the driver gas, with *p*_1_ = 100 kPa and *p*_4_ = 1550 kPa.

For a comparison of the pressure amplitude and the constant pressure time that can be realized by different configurations, the output signal of the DUT was recorded at the same initial conditions using the three configurations, respectively, and the results are shown in Figure 10. Two different settings close to the lower and the upper limits of the operation capacity of the conventional shock tube were tested. As shown in Figure 10a, the initial parameters were *p*_1_ = 100 kPa and *p*_4_ = 800 kPa, and Ar was used in both the driven and driver sections. Figure 10b shows the results at *p*_1_ = 100 kPa and *p*_4_ = 1550 kPa. The driven and the driver gases were Ar and He, respectively. The figure shows that the signal amplitude increased about 15 times and 5 times when using the converging cone and the amplification system, respectively, compared to the conventional configuration. On the other hand, the constant signal time decreased to about 0.2 ms in the case of the amplification system compared to about 1.5 ms when using the conventional shock tube. When using the converging cone, the output signal has a blast profile. Furthermore, a high-frequency oscillation can be seen in the signal when using the amplification system. The frequency of this oscillation increases with signal amplitude, and this oscillation will be discussed in the next section.

**Figure 11:**
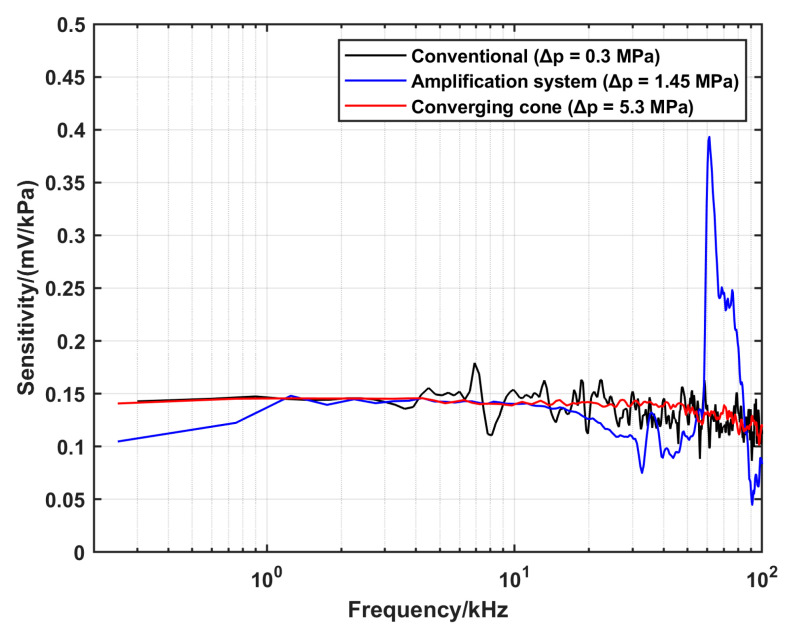
A comparison of the dynamic response of the DUT using the three configurations at the same initial parameters, with Ar as the driven and driver gas, whereby *p*_1_ = 100 kPa and *p*_4_ = 800 kPa.

The DUT was characterized using the three configurations at the same initial conditions, and the corresponding reference pressure and sensitivity were calculated according to the procedures in Section 3. The summary of the settings and the results is displayed in Table 3. The calculated sensitivity as a function of frequency using the respective configurations is shown in Figure 11.

The given value of the sensitivity from the manufacture is 0.145 mV/kPa.

**Figure 12:**
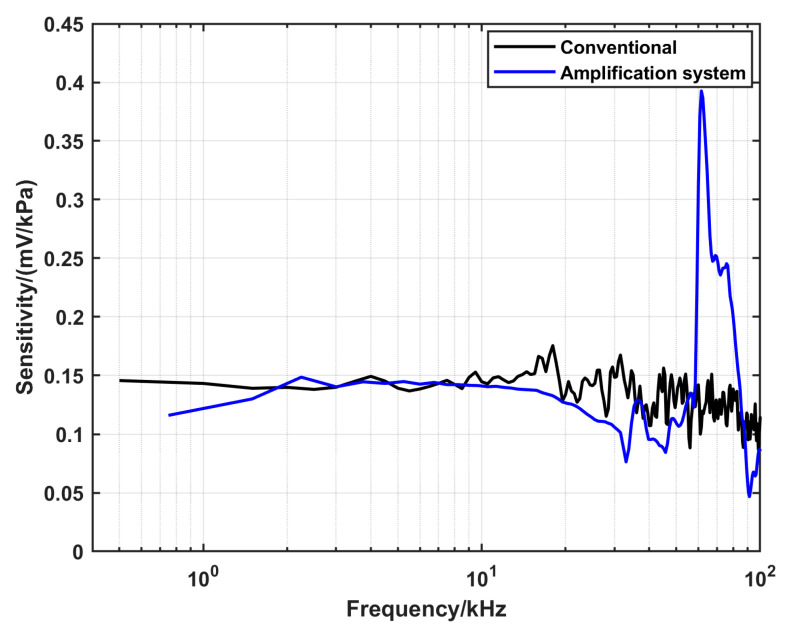
A comparison of the dynamic response of the DUT at a nominal pressure of 1.45 MPa, characterized using the conventional and the amplification system configurations, respectively.

**Figure 13:**
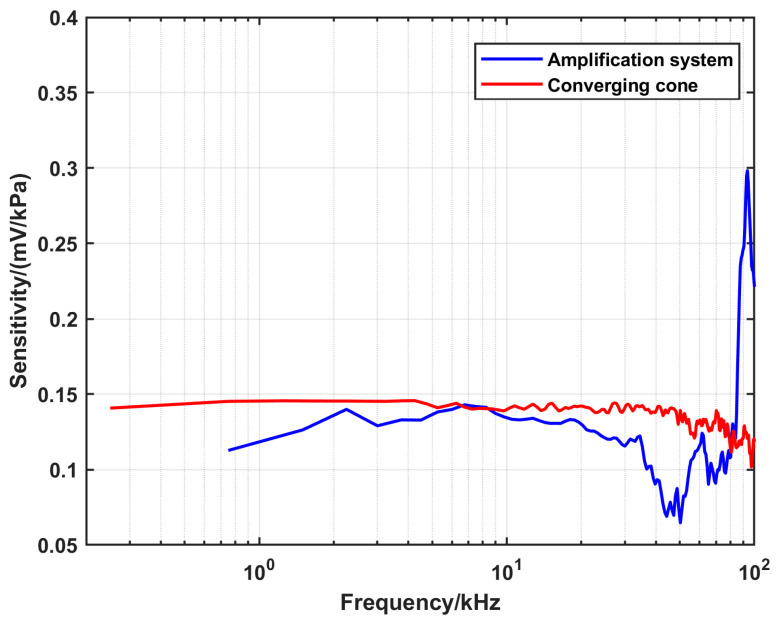
A comparison of the dynamic response of the DUT at a nominal pressure of 5.3 MPa, characterized using the amplification system and the converging cone configurations, respectively.

To validate the configurations developed in this work, the sensitivity of the DUT was evaluated using different configurations. The main assumption was that the DUT behaves as a linear time-invariant system [26]. Therefore, it should have a response function (sensitivity) that is independent of the input signal (the reference pressure). To perform this comparison, nominal pressures that can be realized by using two alternatives were tested. There is an overlap between the upper limit of the conventional shock tube and the lower limit of the amplification system configuration. A pressure amplitude of 1.45 MPa can be realized by the two configurations using different gas conditions in the driver section. He with a pressure of 1550 kPa was used in the case of the conventional shock tube, while Ar with a pressure of 800 kPa was used in the case of the amplification system. The calculated sensitivities corresponding to the two configurations, respectively, are presented in Figure 12. To compare the amplification system and the converging cone, a nominal pressure of 5.3 MPa was tested. For the amplification system, the driver gas was He with a pressure of 1000 kPa, while Ar with a pressure of 800 kPa was used in the case of the converging cone. The corresponding calculated sensitivities are shown in Figure 13.

Figure 12 shows that there is a good overlap between the sensitivities estimated by the two configurations in the range from 2 kHz up to 12 kHz. On the other hand, Figure 13 shows that the sensitivity calculated by the converging cone is uniform up to 40 kHz, while the sensitivity from the amplification system is underestimated in the range from 3 kHz to 4 kHz.

### 4.2. Uncertainty in Sensitivity Calculations Using the Conventional Tube and the Amplification System

The uncertainty budget was evaluated in accordance with JCGM 100:2008 [27]. The uncertainty in *p*_5_ was estimated using the Monte Carlo method. In the Monte Carlo method, *p*_5_ was calculated 10^6^ times according to Equations (1) and (4), with the input parameters independently and randomly distributed, as described in Table 4. The standard deviation of the resulting distribution of *p*_5_ is the standard uncertainty, *u*(*p*_5_).

A detailed description of the uncertainty in each input parameter can be found in reference [10]. The uncertainty in time when using the amplification system was 2.5 μs compared to 0.4 μs in the case of the conventional skock tube to account for the uncertainty due to using two side-wall sensors and the end-wall sensor to calculate the shock wave speed.

The combined standard uncertainty in the step pressure amplitude, *u*(Δ*p*), was calculated according to

(7) $$u\left(∆p\right)=\sqrt{u^{2}\left(p_{5}\right)+u^{2}\left(p_{1}\right)}.$$

The corresponding standared uncertainty in sensitivity $u_{∆p}\left(s\right)$ was calculated as

(8) $$u_{∆p}\left(s\right)=\frac{-V_{sig}}{{\Delta p}^{2}}\times u\left(∆p\right)$$

where $V_{sig}$ is the amplitude of the recorde signal. To estimate the uncertainty in sensitvity due to repetability, three shots were recorded at each nominal pressure level. The standard uncertainty in sensitivity at each frequency due to repeatability $u_{rep}(s)$ was calculated as

(9) $$u_{rep}\left(s\right)=(s_{max}-s_{min})/\sqrt{12}$$

The combined standard uncertainty (*k* = 1) in sensitivity $u_{c}\left(s\right)$ was estimated as

(10) $$u_{c}\left(s\right)=\sqrt{u_{∆p}^{2}\left(s\right)+u_{rep}^{2}\left(s\right)}$$

**Figure 14:**
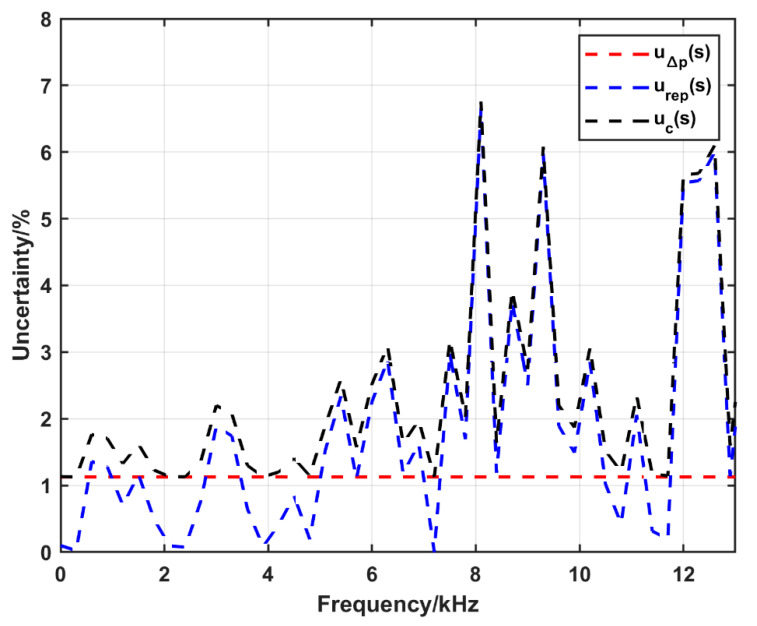
The expanded uncertainty (*k* = 2) in sensitivity calculation up to 13 kHz using the conventional configuration at a nominal pressure of 0.3 MPa, $u_{∆p}(s)$: the uncertainty due to the uncertainty in Δ*p*, $u_{rep}(s)$: the uncertainty due to the repeatability, $u_{c}(s)$: the combined uncertainty.

**Figure 15:**
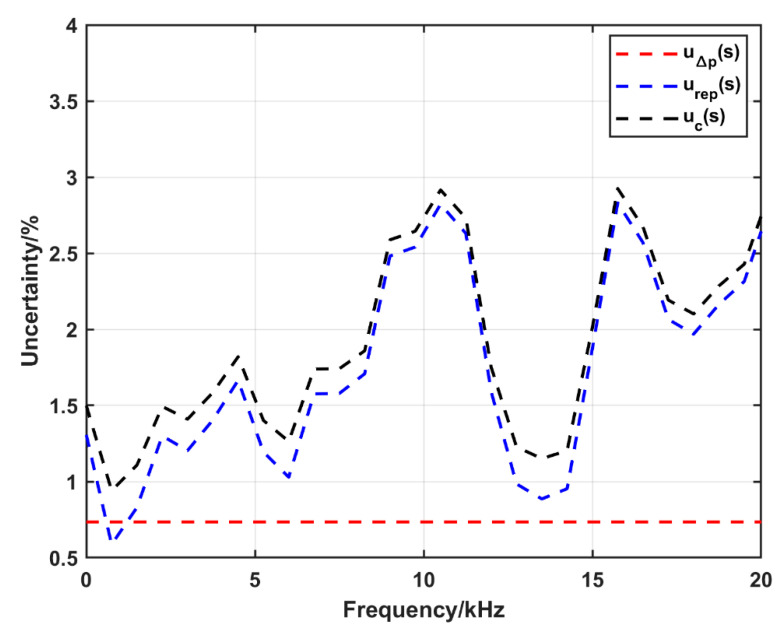
The expanded uncertainty (*k* = 2) in sensitivity calculation up to 20 kHz using the amplification system at a nominal pressure of 1.45 MPa, $u_{∆p}\left(s\right)$: the uncertainty due to the uncertainty in Δ*p*, $u_{rep}(s)$: the uncertainty due to the repeatability, $u_{c}(s)$: the combined uncertainty.

The calculated expanded uncertainty (*k* = 2) as a function of frequency in the case of using the conventional configuration (at nominal pressure of 0.3 MPa) and the amplification system at nominal pressure of 1.45 MPa is shown in Figure 14 and Figure 15, respectively. The figures show that the uncertainty due to the uncertainty in Δ*p* is about 1.1% and 0.7% in the case of the conventional configuration and the amplification system, respectively. In both figures the uncertainty due to repeatability is dominant. In the conventional configuration, the combined uncertainty is about 3%; however, there are some jumps to about 6% at 8 kHz, 9 kHz and 13 kHz. On the other hand, it is less than 3% up to 20 kHz in the amplification system configuration.

### 4.3. Uncertainty in Sensitivity in the Case of the Converging Cone

The uncertainty in the reference pressure was assessed by making additional simulations using input parameter values that are offset from their respective centre value by their uncertainty. The input parameters are, the pressure *p*_2_, particles speed *U*_2_ and temperature *T*_2_ behind the incident shock wave. The uncertainty in these parameters were calculated using the Monte Carlo method. The parameters were calculated10^6^ times using Equations (1)–(3), with the input parameters independently and randomly distributed according to Table 4. The standard deviations of the resulting distribution of *p*_2_, *U*_2_ and *T*_2_ are the standard uncertainty in the parameters $u_{p_{2}}$, $u_{U_{2}}$ and $u_{T_{2}}$. To assess the uncertainty in the reference pressure, the uncertainty was calculated at three different pressure levels: the minimum (5.3 MPa), the maximum (250 MPa) and a middle (150 MPa) pressure that can be realized by the converging cone. At each level, seven simulations were run with the centre value of the three parameters, which were offset from the centre value by the standard uncertainty for the respective parameters in the plus and in the minus directions, respectively. For clarification, the input parameters for the seven runs were (*p*_2_, *U*_2_, *T*_2_), (*p*_2_ + $u_{p_{2}}$, *U*_2_, *T*_2_), (*p*_2_ − ${\;u}_{p_{2}}$, *U*_2_, *T*_2_), (*p*_2_, *U*_2_ + $u_{U_{2}}$, *T*_2_), (*p*_2_, *U*_2_ − ${\;u}_{U_{2}}$, *T*_2_), (*p*_2_, *U*_2_, *T*_2_ + $u_{T_{2}}$) and (*p*_2_, *U*_2_, *T*_2_ − $u_{T_{2}}$), respectively. The relation between the uncertainty in each parameter and the corresponding produced uncertainty in the reference pressure was identified and used for estimating the uncertainty at different pressure levels. Furthermore, the uncertainty due to the uncertainty in cone dimension measurements was assessed. The cone dimensions were measured, and the uncertainty in measurements was used to assess the corresponding uncertainty in the reference pressure, resulting in a relative standard uncertainty of 0.4%. An example of the standard uncertainty in the reference pressure corresponding to the standard uncertainty in the input parameters at a nominal reference pressure of 5.3 MPa is shown in Table 5. The calculated input parameter values were *P*_2_ = 211.8 kPa, *U*_2_ = 154.8 m/s and *T*_2_ = 676.5 K.

**Figure 16:**
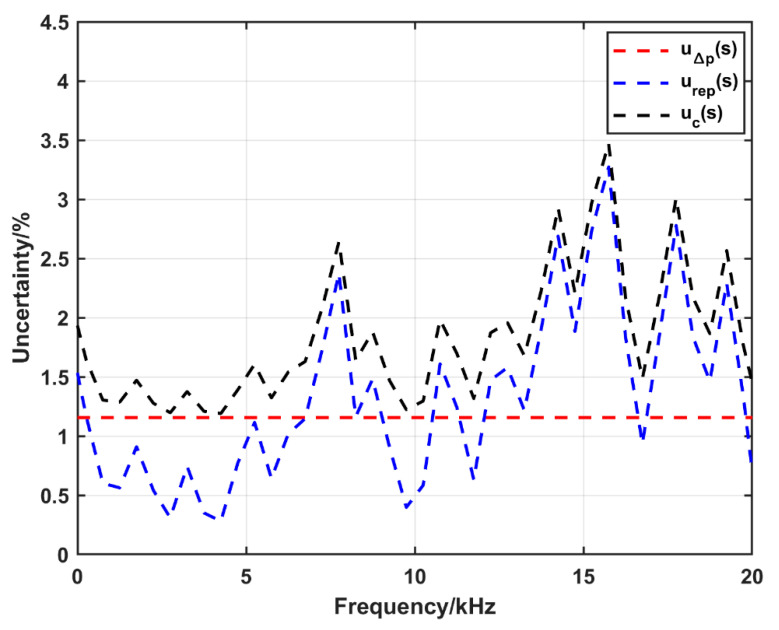
The expanded uncertainty (*k* = 2) in the sensitivity calculation up to 20 kHz using the converging cone at a nominal pressure of 5.3 MPa, $u_{∆p}\left(s\right)$: the uncertainty due to the uncertainty in Δ*p*, $u_{rep}(s)$: the uncertainty due to the repeatability, $u_{c}(s)$: the combined uncertainty.

The combined standard uncertainty in *p*_5_ was calculated as the square root of the summation of the quadratic value of the produced uncertainty corresponding to the uncertainty in different parameters. Then, following Equations (7)–(10), the uncertainty in the calculated sensitivity can be estimated. The calculated expanded uncertainty (*k* = 2) as a function of frequency at a nominal pressure of 5.3 MPa is shown in Figure 16. An uncertainty less than 3.5% up to 20 kHz can be seen in the figure at this pressure level.

## 5. Discussion

The results in the previous section show that the three configurations complete each other. The capabilities and the shortages in each configuration will be discussed.

For the conventional shock tube, the upper pressure limit is restricted by the maximum static pressure that the shock tube can safely withstand. The driver section is equipped with a safety valve that opens automatically when *p*_4_ exceeds 1.7 MPa. The constant pressure time is about 2–3 ms, which extends the lower frequency limit of the shock tube to 0.3 kHz. The signal-to-noise ratio is relatively low at high frequencies, and variations between different shots and frequency can be seen in the uncertainties due to repeatability. Using the well-established analytical solution to calculate the reference pressure in this configuration emphasizes SI traceability.

In the case of the amplification system, this configuration fills the gap in the pressure amplitude between the conventional configuration and the converging cone. The pressure constant time is relatively short, which limits the lower frequency that can be realized when using this configuration; however, it is long enough to use the analytical solution for the reference pressure calculation. A drawback of this configuration is that for calculating the speed of the shock wave, two side-wall sensors and the end-wall sensor were used. The variation in the interaction direction of the shock front parallel/normal in relation to the sensor may result in less accuracy in estimating the shock speed. Furthermore, if these sensors have different latencies, this also can lead to errors in the measurements. In this study, the end-wall sensor was the same model as the two side-wall sensors to minimize this risk. Additionally, the uncertainty in time was set to a higher value compared to using the conventional configuration. To overcome this in the future work, a new extension tube that is long enough to mount three side-wall sensors will be manufactured. This will be achieved at the expense of the pressure amplitude since the amplitude decreases with the propagation distance of the shock due to energy loses. Moreover, in the dynamic response of the DUT, there is a peak around 60 kHz at 1.45 MPa, which shifted to a higher frequency of 90 kHz at 5.3 MPa, corresponding to the oscillations seen in the time domain (see Figure 10). This peak limits the upper frequency that currently can be realized by this system. The reason of this oscillation is the generation of a standing wave in the gas medium. Which is evident from the fact that the frequency of the oscillation scales with the speed of sound propagation, which depends on the temperature (*T*_5_) of the reflected shock wave. Moreover, it is localized around the DUT since it is not recorded by the side-wall sensors. The cause of this standing wave is a defect in the backplate design. There is a groove with a depth of 2.5 mm for mounting an O-ring used in another experiment, as shown in Figure 8c. This groove, which acts as a pipe with a length of 2.5 mm, has one fixed end and one free end. For the standing wave condition with this geometry, the calculated frequency is equal to the frequency of the peak seen in Figure 12 and Figure 13 when using the corresponding sound speed, which emphasizes our clarification. This defect in the backplate design will be avoided in the future design.

In the converging cone configuration, an amplification of the pressure amplitude of about 15 times can be achieved by increasing the capability of the shock tube. However, because of the blast profile of the reference pressure, the analytical solution is no longer applicable, and numerical simulation was mandatory for the reference pressure calculation. Despite using a numerical simulation, the calculated DUT sensitivity was in a good agreement with that estimated using the other configurations and with the value given from the manufacture. One drawback of this configuration is that the DUT should have a large bandwidth and be fast enough to record the blast signal. Furthermore, calculating the reference pressure is time-consuming, as the estimated time for one simulation is about four hours.

From the uncertainty calculations for the three configurations, it is seen that the uncertainty in sensitivity is dominated by the uncertainty due to repeatability. In all configurations, the contribution from the uncertainty in the reference pressure calculation is less than 1.5%.

## 6. Conclusions

The shock tube at RISE, the National Metrology Institute of Sweden, was developed to extend its capability to the high-pressure regime. The shock tube was modified to be operated in three different configurations: conventional, with an amplification system and with a converging cone. The three configurations complement each other, allowing them to cover the pressure range from 0.1 to 25 MPa and frequencies from 0.5 kHz to 500 kHz. The limitations of this study due to the dependence on numerical simulation in the case of the converging cone will be minimized by validating the simulation results. A comparison with different facilities will be carried out in the future. Furthermore, the measurements error that may result from using sensors with different latencies in calculating the shock speed in the case of the amplification system configuration will be suppressed by manufacturing a new extension tube which is long enough for mounting three side-wall sensors. The novelty of this work lies in the development of effective methods of pressure amplification using new technologies. Using the new configurations of the shock tube results in improvements of the accuracy of dynamic pressure calibration, which in turn can lead to significant improvements in areas such as automotive and aerospace engineering.

## Figures and Tables

**Table 1:** A list of the initial and boundary conditions of the parameters used in the simulation.

	Driven Section		Driver Section		
	IC	BC Walls	IC	BC Walls	BC Inlet
*P* (kPa)	100	zeroGradient	Calculated [25]	zeroGradient	waveTransmissive uniform calculated [25]
*U* (m/s)	0	fixedValue (0, 0, 0)	Calculated [25]	fixedValue (0, 0, 0)	zeroGradient
*T* (K)	294.15	fixedValue 294.15	Calculated [25]	fixedValue calculated [25]	zeroGradient

**Table 2:** The characteristics of the four configurations of the shock tube tested in this study.

Configuration	Conventional	Converging Cone	Amplification System (First Trial)	Amplification System
Reference pressure profile	Step-like	Blast	Blast	Step-like
Reference pressure amplitude (MPa)	0.1–1.5	5–25	3–12	1–7
Constant pressure time (ms)	2–3	≤0.005	0.02	0.2–0.3
Calculation of the reference pressure	Analytical solution	Numerical simulation	Numerical simulation	Analytical solution

**Table 3:** A summary of the settings and results from the characterization of the DUT using the three configurations.

	Driven Gas	Driver Gas	*p*_1_ (kPa)	*p*_4_ (kPa)	Δ*p* (MPa)	Average Sensitivity (mV/kPa)
Conventional	Ar	Ar	100	800	0.30	0.145 (up to 20 kHz)
Amplification system	Ar	Ar	100	800	1.45	0.142 (from 2 kHz to 13 kHz)
Converging cone	Ar	Ar	100	800	5.30	0.142 (up to 20 kHz)

**Table 4:** List of the input parameters in the step pressure calculation and their corresponding standard uncertainty.

**Parameter**	**Standard Uncertainty, *k* = 1**	**Distribution**
Temperature (K)	0.6	Normal
Time (s)	0.4 × 10^−6^ (conventional) 2.5 × 10^−6^ (amplification system)	Normal
Side-wall sensors positions (m)	10^−4^	Normal
Specific heat ratio *γ*_1_	10^−4^	Normal
Initial driven pressure *p*_1_ (Pa)	100	Normal

**Table 5:** An example of the standard uncertainty in the reference pressure corresponding to the uncertainty in input parameters at a nominal reference pressure of 5.3 MPa.

The Input Parameters	The Standard Uncertainty in the Input Parameters (*k* = 1)	The Corresponding Standard Uncertainty in *p*_5_ (*k* = 1) (kPa)
*P*_2_ (kPa)	0.6	18.2
*U*_2_ (m/s)	0.4	11.0
*T*_2_ (K)	0.5	0.1
Cone dimensions (m)	5 × 10^−5^	0.4% of *p*_5_ (22 kPa)

## Data Availability

Data are contained within the article.
